# Self-Induced Euglycemic Diabetic Ketoacidosis: When to Stop the Drip

**DOI:** 10.7759/cureus.21768

**Published:** 2022-01-31

**Authors:** Marc T Zughaib, Kunjal Patel, Mariola Leka, Saif Affas

**Affiliations:** 1 Internal Medicine, Ascension Providence Hospital, Southfield, USA

**Keywords:** adult internal medicine, endocrinology and diabetes, diabetes treatment, sglt-2 inhibitor, euglycemic dka

## Abstract

Diabetic ketoacidosis (DKA) is a well-known, serious complication that many patients with type 1 and 2 diabetes face due to either a relative or absolute insulin deficiency. Sodium-glucose cotransporter 2 (SGLT-2) inhibitors have gained increased popularity due to their diabetic, cardiovascular, and renal benefits. An associated complication of SGLT2 inhibitors is euglycemic DKA.

A 56-year-old male with a history of type 2 diabetes mellitus and peripheral neuropathy presented with right foot pain secondary to a diabetic foot ulcer. The ulcer was present for one year, but the patient noticed increased pain and purulent discharge over the three days prior to presentation. While being treated inpatient for the foot ulcers, the patient repeatedly refused to receive standard hospital diabetes management of insulin injections. He instead insisted to take his home medications against medical advice, which were metformin and Glyxambi® (empagliflozin/linagliptin, Boehringer Ingelheim, Ingelheim am Rhein, Germany). His diabetic foot ulcer was medically managed with IV antibiotics.

On day 4 of admission, his anion gap increased to 23 mEq/L, and serum bicarbonate (CO2) decreased to 8 mEq/L, raising concerns of diabetic ketoacidosis. His glucose was 141 mg/dL, his beta-hydroxybutyrate was high at 5.5 mmol/L. An arterial blood gas (ABG) test demonstrated anion gap metabolic acidosis with secondary respiratory alkalosis. A urinalysis demonstrated glucose 1000 mg/dL and ketones of 150 mg/dL. The patient was diagnosed with euglycemic DKA. Due to the patient having normal glucose levels, an insulin drip and a 5% dextrose with 0.45% normal saline drip were started. Basic metabolic profiles were ordered every four hours, with glucose checks every hour. Once the anion gap was closed and his urinary ketones disappeared, the patient transitioned to subcutaneous insulin. He was able to be discharged home with basal subcutaneous insulin and metformin with instructions to avoid SGLT2 inhibitors in the future.

Unfortunately, there are currently no guidelines from endocrinology or internal medicine societies regarding the management of euglycemic DKA. As the typical DKA diagnostic criteria of elevated blood glucose level are not present, it is easy to overlook euglycemic DKA. As these SGLT2 inhibitors become more prevalent, careful monitoring of all potential side effects as well as the contraindications are prudent to successful management of complex disease states.

## Introduction

Diabetic ketoacidosis (DKA) is a well-known, serious complication that many patients with type 1 and 2 diabetes face due to either a relative or absolute insulin deficiency [1]. DKA is a medical emergency with the following diagnostic criteria: hyperglycemia with blood glucose >250 mg/dL, anion gap metabolic acidosis with serum bicarbonate <18 mEq/L, and arterial pH <7.3, with ketones present in urine and serum [1, 2]. Generally, DKA presents for a variety of reasons some of which include medication non-compliance, infections, infarctions, and undiagnosed diabetes mellitus (DM). Due to life-threatening complications of untreated severe hyperglycemia, prompt recognition and treatment of DKA are warranted. 

Sodium-glucose cotransporter 2 (SGLT2) inhibitors have gained increased popularity due to their cardiovascular and renal benefits. Evidence has dramatically increased over the past several years based on recent studies, including the EMPAREG OUTCOME (Empagliflozin Cardiovascular Outcome Event Trial in T2D Patients-Remove Excess Glucose) and the CANVAS (Canagliflozin Cardiovascular Assessment Study) Program (comprising the CANVAS and CANVAS-R trials) have demonstrated significant reductions in major adverse cardiac events (MACE) in patients randomized to receive SGLT2 inhibitor therapy compared with placebo [3, 4]. Additionally, several studies have shown reduced hospitalizations for patients with heart failure with reduced or preserved ejection fractions, as well as slowing the progression of chronic kidney disease [5-10].

The US Food and Drug Administration (FDA) and European Medicines Agency (EMA) describe diabetic ketoacidosis with SGLT2 inhibitors with normal blood sugar levels [11, 12]. The incidence of euglycemic DKA with SGLT2 inhibitors was described as 0.1%, rates ranging from 0.16 to 0.76 events per 1000 patient-years in patients with type 2 diabetes [13, 14]. These patients typically present with normal or only mildly elevated blood glucose levels (<13.9 mmol/L, <250 mg/dL). Due to this absence of significant hyperglycemia, there is generally a delay in diagnosis. Here we present a complicated case of self-induced euglycemic DKA in a hospitalized patient.

## Case presentation

A 56-year-old male with a past medical history of type 2 diabetes mellitus and peripheral neuropathy presented with right foot pain secondary to a diabetic foot ulcer. The ulcer was present for 1 year, but the patient noticed increased pain and purulent discharge over the 3 days prior to presentation. There was a second ulceration on the patient’s right lateral fourth toe expressing purulent drainage as well. His diabetic regimen included metformin 1000 mg BID and Glyxambi (combination empagliflozin and linagliptin; Boehringer Ingelheim, Ingelheim am Rhein, Germany), with a hemoglobin A1c of 11.2%.

**Figure 1:**
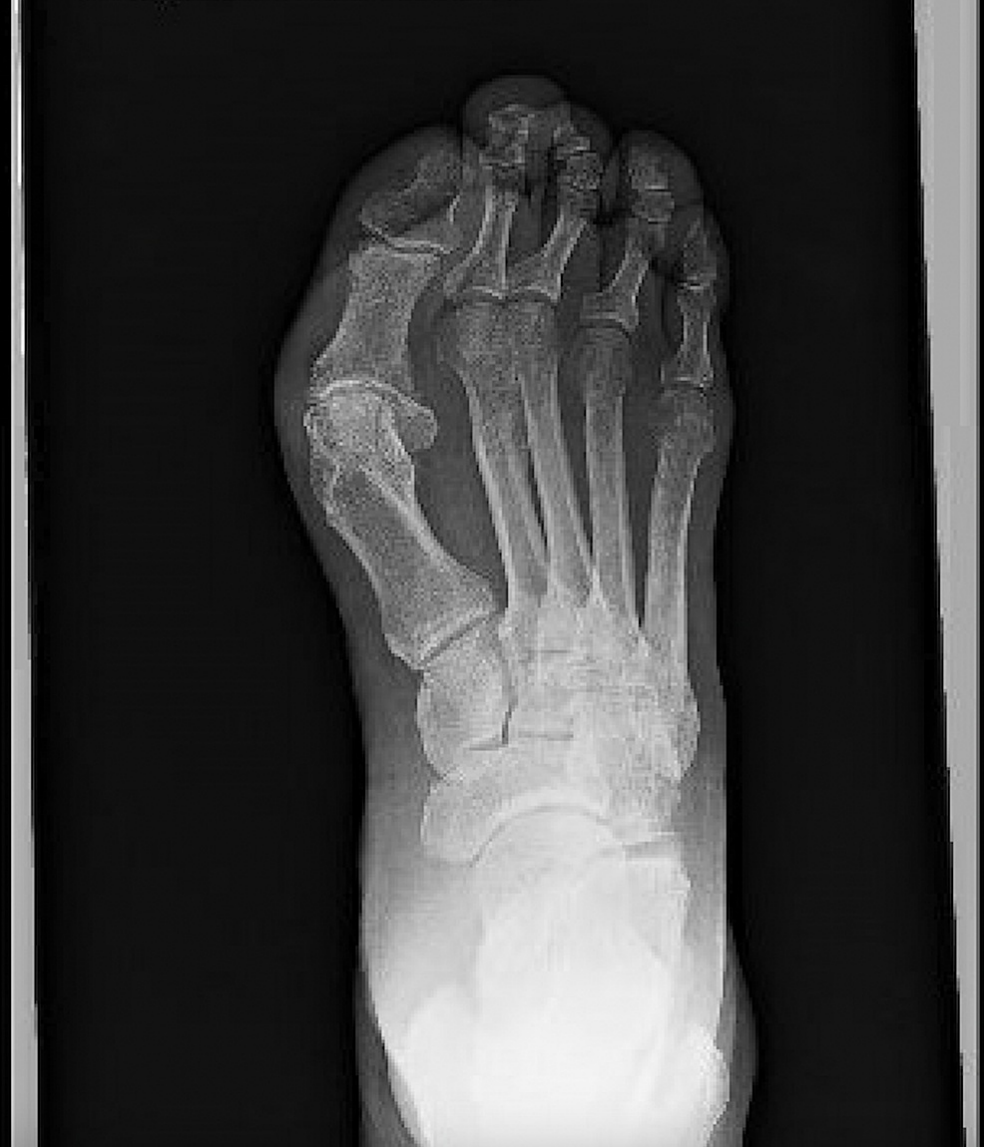
X-ray of the right foot which demonstrated severe degenerative changes of the first metatarsal phalangeal joint without evidence of acute osteomyelitis.

**Figure 2:**
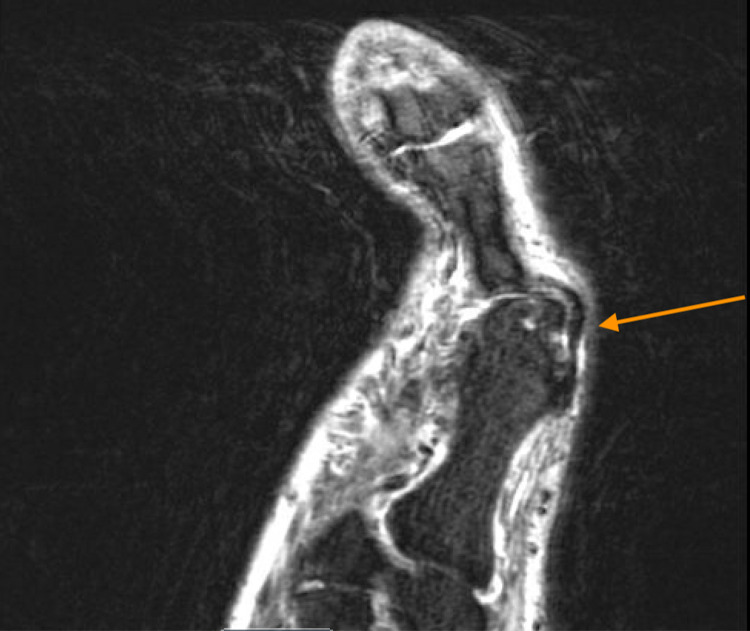
MRI foot demonstrating cellulitic inflammatory changes without osteomyelitis. Orange arrow points at the changes described above.

In the emergency department, the patient was afebrile and vitals were stable. On physical examination, there was a full-thickness ulceration with purulent drainage which probed to the bone. There was streaking lymphangitis and cellulitis present as well. His white blood cell count was 7,000 per microliter. Erythrocyte sedimentation rate and C-reactive protein were elevated at 40 mm/hr and 126 mg/L, respectively. The probe to bone test was positive. An X-ray of the foot was performed which demonstrated severe degenerative changes of the first metatarsal phalangeal joint with mild soft tissue swelling over the dorsum of the foot without radiographic evidence of acute osteomyelitis (Figure 1). An MRI of the foot was ordered, which suggested cellulitic inflammatory changes without osteomyelitis (Figure 2). Blood and wound cultures were collected, and the patient was started on cefepime and vancomycin.

While being treated inpatient for the foot ulcers, the patient adamantly and repeatedly refused to receive standard hospital diabetes management with short- and long-acting insulin. This was continuously offered to the patient to help control his blood glucose levels, but the patient instead stated he brought his home medications, metformin and Glyxambi. He continued taking them against the advice of the medical team. During the hospitalization, his blood glucose levels remained within the acceptable range. Taking his home medications without the supervision of the primary team or nursing staff, there was concern the patient may have been taking larger doses of the medications in order to keep his blood glucose levels within the normal limits. 

On day 4 of admission, his anion gap went from 16 to 23 mEq/L and his CO2 went from 12 to 8 mEq/L, raising concerns of diabetic ketoacidosis. While the glucose was normal at 141 mg/dL, his beta-hydroxybutyrate was elevated at 5.5 mmol/L. An arterial blood gas (ABG) test demonstrated an anion gap metabolic acidosis with secondary respiratory alkalosis, with pH 7.23, partial pressure of carbon dioxide (PCO2) 15 mmHg, partial pressure of oxygen (PO2) 108 mmHg, bicarbonate (HCO3) 6.3 mmol/L. The patient's urinalysis demonstrated a glucose of 1000 mg/dL and an elevated ketone level of 150 mg/dL. Once the diagnosis of euglycemic DKA was made, the patient was made NPO and started on an insulin drip with 5% dextrose with 0.45% normal saline at 200 cc/hr. Blood glucose checks were ordered every hour along with a basic metabolic panel (BMP) every 4 hours per DKA management guidelines.

Once the anion gap was resolved the next day, the patient was transitioned to subcutaneous insulin and was discharged home with basal insulin and metformin. The patient was also instructed he will likely need to avoid SGLT2 inhibitors in the future. 

## Discussion

SGLT2 inhibitors are a relatively newer class of oral non-insulin hypoglycemics that are gaining popularity. Their popularity is backed by strong evidence from robust randomized controlled trials [3, 4, 15-19]. SGLT2 inhibitors' mechanism of glucosuria is a result of decreasing the normal renal threshold for reabsorption of glucose from 180 mg/dL to as low as 40 to 120 mg/dL [20]. In SGLT2-treated type 2 diabetic patients with euglycemic DKA, there is a lower insulin-to-glucagon ratio due to this increased glucosuria which stimulates lipolysis. The lipolysis augments free fatty acid delivery to the liver, resulting in mild stimulation of ketogenesis [21]. 

The side effects of SGLT2 inhibitors have been well established through recent landmark clinical trials. The most well-known adverse effect is the higher risk of genital infections. These trials have demonstrated conflicting evidence regarding rates of lower extremity amputations and ulcerations [3, 4]. Despite conflicting results, there are warnings associated with initiating therapy with SGLT2 inhibitors while patients have active diabetic foot wounds, prior amputations, or significant peripheral arterial disease based on the American College of Cardiology (ACC) guidelines [22]. 

There were several predisposing conditions that could have incited out patient’s episode of euglycemic DKA. Uncontrolled diabetes with a hemoglobin A1c above 11%, chronic and severe active foot ulceration/osteomyelitis, and dietary calorie restrictions during the inpatient setting may have contributed to the “perfect storm.” Unfortunately, there are currently no guidelines from endocrinology or internal medicine societies regarding the management of SGLT2 inhibitor-induced euglycemic DKA. In 2016, the American Association of Clinical Endocrinologists (AACE) and the American College of Endocrinology (ACE) released a position statement on the relationship between SGLT2 inhibitors and DKA. Their management recommendation suggested stopping the SGLT2 inhibitor immediately followed by initiation of DKA protocol, including fluids, insulin, and "other standard interventions as described elsewhere" [23]. There have been recommendations based on the management of DKA reported in review articles, which led our treatment protocol [1, 14]. We also obtained expert opinions from current endocrinology attending physicians and fellows regarding the management of euglycemic DKA. The most agreed-upon management included starting the patient on an insulin drip with D5 ½ normal saline. Our patient adamantly refused inpatient insulin treatment/injections for the first several days of admission and would only take his home oral regimen. Despite an HbA1c of 12%, his glucose levels were between 120-150 gm/dL. This raised suspicion of non-compliance in the outpatient setting or the patient taking extra doses of these medications while hospitalized. Since the patient was euglycemic, we believed that monitoring the resolution of ketones in the urine along with a closed anion gap were important to guide the transition from the insulin drip to subcutaneous insulin. 

## Conclusions

As described in the case presented above, multiple factors contributed to a relative insulin deficiency and euglycemic DKA. Increased stressors (i.e. active foot ulceration) with increased urinary excretion of glucose with SGLT2 inhibitors can lead to increased lipolysis and ketogenesis. In the inpatient setting where oral intake is not the same as the outpatient setting, decreased oral intake can further promote the relative insulin-deficient state of SGLT2 inhibitors and lead to euglycemic DKA. Since the typical DKA diagnostic criteria of elevated blood glucose level are not present, it is easy to overlook euglycemic DKA. As these SGLT2 inhibitors become more commonly prescribed and prevalent, careful monitoring of all potential side effects as well as the contraindications are prudent to successful management of complex disease states.
